# Imaging in a Pandemic: How Lack of Intravenous Contrast for Computed Tomography Affects Emergency Department Throughput

**DOI:** 10.5811/westjem.18515

**Published:** 2024-03-29

**Authors:** Wayne A. Martini, Clinton E. Jokerst, Nicole Hodgson, Andrej Urumov

**Affiliations:** * Mayo Clinic Arizona, Department of Emergency Medicine, Phoenix, Arizona; † Mayo Clinic Arizona, Department of Radiology, Phoenix, Arizona

## Abstract

**Introduction:**

During the coronavirus 2019 pandemic, hospitals in the United States experienced a shortage of contrast agent, much of which is manufactured in China. As a result, there was a significantly decreased amount of intravenous (IV) contrast available. We sought to determine the effect of restricting the use of IV contrast on emergency department (ED) length of stay (LOS).

**Methods:**

We conducted a single-institution, retrospective cohort study on adult patients presenting with abdominal pain to the ED from March 7–July 5, 2022. Of 26,122 patient encounters reviewed, 3,028 (11.6%) included abdominopelvic CT with a complaint including “abdominal pain.” We excluded patients with outside imaging and non-ED scans. Routine IV contrast agent was administered to approximately 74.6% of patients between March 7–May 6, 2022, when we altered usage guidelines due to a nationwide shortage. Between May 6–July 5, 2022, 32.8% of patients received IV contrast after institutional recommendations were made to limit contrast use. We compared patient demographics and clinical characteristics between groups with chi-square test for frequency data. We analyzed ED LOS with nonparametric Wilcoxon rank-sum test for continuous measures with focus before and after new ED protocols. We also used statistical process control charts and plotted the 1, 2 and 3 sigma control limits to visualize the variation in ED LOS over time. The charts include the average (mean) of the data and upper and lower control limits, corresponding to the number of standard deviations away from the mean.

**Results:**

After use of routine IV contrast was discontinued, ED LOS (229.0 vs 212.5 minutes, *P* = <0.001) declined by 16.5 minutes (95% confidence interval −10, −22).

**Conclusion:**

Intravenous contrast adds significantly to ED LOS. Decreased use of routine IV contrast in the ED accelerates time to CT completion. A policy change to limit IV contrast during a national shortage significantly decreased ED LOS.

Population Health Research Capsule What do we already know about this issue?

*Intravenous contrast is used to highlight differences between soft tissues that would otherwise look the same.*

What was the research question?

*Does decreased use of contrast for computed tomography (CT) improve ED length of stay (LOS)?*

What was the major finding of the study?

*If there is a shortage of IV contrast for CT, using contrast on fewer patients may improve patient throughput due to shortened LOS.*

How does this improve population health?

*If there is a shortage of IV contrast for CT, using less contrast may improve patient throughput due to shortened LOS.*

## INTRODUCTION

Abdominopelvic computed tomography (CT) is routinely ordered from the emergency department (ED) to evaluate for abdominal pain.^1^ Historically, IV contrast has been used to highlight differences between soft tissues that would otherwise look the same. Intravenous (IV) contrast for CT is often sourced from overseas, and current estimates are that about half of hospitals in the United States get most of their IV contrast agent from GE Healthcare. Much of the contrast dye is manufactured at GE’s plant in Shanghai. During the COVID-19 related lockdowns in China the plant was closed or operating at reduced capacity for weeks. As a result, many hospitals had a significantly decreased supply of IV contrast, which forced them to decrease utilization by up to 80%.

Anticipating continued deficiencies in the supply of IV contrast, Mayo Clinic Arizona in May 2022 initiated critical protocols to limit contrast use to potentially life-threatening conditions. This decreased utilization within the ED created a unique circumstance in which we had the opportunity to explore the theoretical benefit of omitting IV contrast material from routine ED abdominopelvic CT to determine whether it would significantly decrease ED length of stay (LOS), which in our institution we measure as the patient’s total time in the department. Length of stay is a benchmark used by the Centers for Medicare and Medicaid Services as a hospital quality metric.^2^ Additionally, shortened duration of LOS has been shown to decrease the rate that patients leave against medical advice, while increasing patient satisfaction, and potentially improving treatment outcomes.^3^

## METHODS

Prior to the contrast shortage alert, the IV contrast agent iohexol was routinely administered to ED patients in conjunction with CT examinations of the abdomen and pelvis. Starting May 6, 2022, our ED in collaboration with the radiology department agreed to discontinue IV contrast material for routine CT except in two specific scenarios: patients requiring abdominal imaging who had a body mass index (BMI) <25; and patients with a BMI >25 in whom there was an acute, time-dependent concern that required IV contrast to further diagnose.

We designated the “before intervention period” as the 60 days prior to May 6, and the “after intervention period” as the 60 days after May 6. Since the study was focused on process rather than patients, the normal requirement for institutional review board oversight was waived. We included in the study patients who presented to the ED with abdominal pain and underwent abdominopelvic CT at the discretion of the treating attending emergency physician. The primary outcome was ED LOS, which was defined as the length of time between when the patient registered for care in the ED and the time of ED disposition (admit or discharge time).

Median and interquartile range (IQR) values were expressed for all continuous measures between groups (before vs after periods). We compared patient demographics and clinical characteristics between groups with chi-square test for frequency data and nonparametric Wilcoxon rank-sum test for continuous measures. The primary outcome was ED LOS. We analyzed data using statistical process control charts (with 1, 2 and 3 sigma control limits), and we adjusted confidence limits using an XmR chart, which helps to determine how a process changes over time. The XmR control chart is recommended for LOS and real-world ED operational data.^4^ Control charts were run for all scans and separated out by contrast administration for both time periods. *P*-values <0.05 were considered statistically significant. We used R version 4.1.2 ggQC package (RStudio, Boston, MA) for statistical analysis.

## RESULTS

There were 26,122 patient encounters within the study period, of which 3,028 (11.6%) met the study criteria: complaint at triage of abdominal pain; age >18; and indications for CT exclusive of ureterolithiasis. Median age was 60 years (IQR 40–72). Following protocol change, there was a 41.8% absolute decrease in abdominopelvic CT studies that used IV contrast : 74.6% (1,120/1,502) before vs 32.8% (500/11,526) after; *P* < 0.001). There was also a 16.5-minute decrease in LOS (95% confidence interval −10, −22) from 229.0 vs 212.5 minutes (Table).

**Table.** Before vs after restrictions on use of intravenous contrast for abdominal/pelvic computed tomography, demonstrating the impact on length of stay in the emergency department.

	Before (n = 1,502)	After (n = 1,526)	Total (N = 3,028)	*P*-value
Total LOS (minutes)				<0.001
Mean (SD)	239.6 (89.2)	226.3 (96.3)	232.9 (93.1)	
Median	229.0	212.5	220.0	
Q1, Q3	176.0, 293.0	156.3, 281.0	167.0, 287.0	
Range	13.0 – 672.0	7.0 – 877.0	7.0 – 877.0	
Contrast received				<0.001
No	382 (25.4%)	1026 (67.2%)	1408 (46.5%)	
Yes	1120 (74.6%)	500 (32.8%)	1620 (53.5%)	

*LOS*, length of stay; *Q*, quartile; *SD*, standard deviation.

## DISCUSSION

We believe that radiology can significantly impact patient throughput.^5^ Our findings suggest that decreased use of IV contrast in non-essential imaging of the abdomen and pelvis is associated with a decrease in ED LOS, thereby improving ED throughput. While 16.5 minutes may seem like a brief length of time, in this patient sampling it reduced LOS by about 7.2% (229 vs 212.5 minutes) and reduced aggregate LOS by a combined total of 420 hours over the course of nine weeks. This time savings multiplied by the millions of patients who present to the ED annually for abdominal pain can translate into a large magnitude of time saved, further decreasing the strain on the ED and potentially improving patient satisfaction.^6^ As our study was performed at an institution with high nursing staff levels (2–3 patients per nurse) and tech ratios (six patients per tech), thereby optimizing time to IV access and kidney function test results, we hypothesize there would be even more pronounced improvement in LOS at facilities that are short staffed.

Additionally, discontinuation of contrast can help to reduce incidence of need for IV-line placement, and the risk for allergy/anaphylaxis. In conversations with the radioloy department, the radiologists emphasized that they felt more confidence in the accuracy of their diagnoses with the use of contrast and that non-urgent findings such as carcinoma would more likely be missed without contrast. They suggested that reduced use of IV contrast would be appropriate in settings where artificial intelligence has improved pathology recognition or in the event of another shortage of contrast agent. More research will be needed to investigate the clinical effect of discontinuing IV contrast in this setting.

## LIMITATIONS

There are several limitations to our study. After reviewing the data at other sites (Mayo Clinic Rochester, Mayo Clinic Florida, and Mayo Clinic Health System) we decided to make this a single-center study as other sites were not affected in the same way by the shortage, and they had a more gradual rollout of IV contrast restrictions. While we noted a reduction in LOS, we were unable to clearly parse out whether it resulted from decreased need for IV access and lab results, or decreased time in radiology department. Additionally, our study encompassed a limited time frame of only about 60 days, after which IV contrast agent became more available. Lastly, more research is needed to further analyze the potential need for repeat imaging or possible return visits to the ED as a result of not using IV contrast.

## CONCLUSION

In this single-center study, we found that an institutional policy change reducing the use of contrast in abdominal-pelvic CT during the COVID-19 pandemic was significantly associated with shorter length of stay in the ED.
